# Primary Serous Cystadenocarcinoma of the Spleen

**DOI:** 10.7759/cureus.55165

**Published:** 2024-02-28

**Authors:** Peter Sciberras, Amey Baitule, Alexander Wilkins, Alex Buchanan

**Affiliations:** 1 Surgery, Hull University Teaching Hospitals NHS Trust, Hull, GBR; 2 Pathology, Hull University Teaching Hospitals NHS Trust, Hull, GBR

**Keywords:** hepatopancreatobiliary, surgery, serous cystadenocarcinoma, cancer, spleen

## Abstract

Tumours of the spleen are uncommon, and most are metastases from primaries in other organs. Primary splenic malignancies are subdivided into two main groups: lymphoid and non-lymphoid. Primary splenic cystadenocarcinomas are extremely rare, and only reports of the mucinous variant exist. We present the case of a female in her eighth decade of life who was found to have an incidental complex splenic mass with a cystic component, which showed an interval increase in size on serial imaging. After further investigation, including positron emission tomography (PET), endoscopic ultrasound (EUS), and laparoscopy, she successfully underwent distal pancreatectomy, splenectomy, and partial gastrectomy for a suspected locally invasive pancreatic malignancy. Histology and immunohistochemical analyses were consistent with the first recorded case of primary serous cystadenocarcinoma of the spleen in the literature.

## Introduction

Tumours of the spleen are uncommon, and the majority are secondary to primaries elsewhere. The two main subdivisions of primary splenic malignancy are lymphoid and non-lymphoid [1]. Primary splenic cystadenocarcinomas are extremely rare, and only tumours of the mucinous subtype have been described. Mucinous cystic neoplasms in other intra-abdominal organs have common clinicopathological profiles and are grouped together as ‘extraovarian’ [2]. Splenic infiltration from primary cystadenocarcinomas in other organs normally occurs in the context of widespread metastatic disease, although reports of solitary metastatic lesions developing in the spleen from neoplasms of other organs exist [3]. Serous cystic tumours most commonly arise in the ovaries, and serous cystadenocarcinomas make up over 90% of ovarian malignancies [4]. We hereby report a case of primary serous cystadenocarcinoma of the spleen.

This article was previously presented as a poster at the 26th Association of Upper Gastrointestinal Surgery of Great Britain and Ireland (AUGIS) Annual Scientific Meeting in Oxford, England, on September 28, 2023.

## Case presentation

A Caucasian female in her 80s with well-controlled hypertension, hypothyroidism, and emphysema initially presented in August 2018 with right iliac fossa pain. Computed tomography (CT) did not find a cause for her symptoms. However, an incidental 2.5 cm ill-defined low-attenuation lesion in the splenic hilum was detected. The patient was discharged with a plan for outpatient gastrointestinal endoscopy, which was unremarkable, and follow-up CT three and 12 months later. The lesion was further characterised as likely representing a benign splenic haemangioma, which remained unchanged in size on repeat imaging.

**Figure 1:**
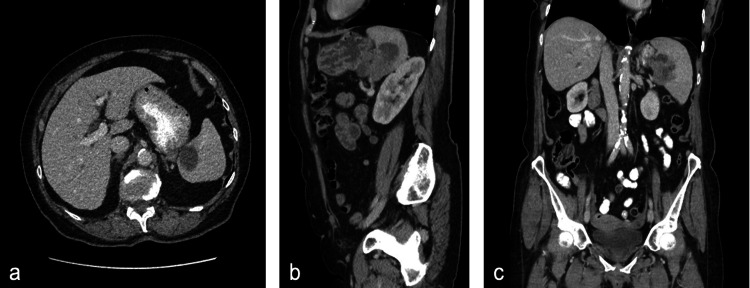
Axial (a), sagittal (b), and coronal (c) planes of CT showing a complex mass with a cystic component arising from the splenic hilum.

The patient then re-presented in May 2022, this time with left iliac fossa pain. The splenic mass showed interval enlargement compared to previous imaging obtained in July 2019 (Figure 1), and the patient was referred to the regional hepatopancreatobiliary (HPB) multidisciplinary team (MDT) meeting for discussion. Magnetic resonance imaging (MRI) confirmed the presence of a 5-cm complex splenic mass with a cystic component, projecting beyond the limits of the spleen towards the fundus of the stomach and the tail of the pancreas. Her normal inflammatory markers made an abscess unlikely. Fluorodeoxyglucose (FDG)-positron emission tomography (PET) identified an intensely FDG-avid 5.7 cm splenic hilum lesion suspicious of lymphoma. Cytology obtained via endoscopic ultrasound (EUS) demonstrated moderate-to-poorly differentiated adenocarcinoma, likely from a pancreatic primary. Her American Society of Anesthesiologists (ASA) grade was two, with an initial Eastern Cooperative Oncology Group (ECOG) performance status of one. However, her cardiopulmonary exercise test (CPEX) was inconclusive, and she was deemed high-risk. After counselling, she decided to undergo surgery for a presumed locally invasive tail of pancreas malignancy. Up until surgery, she still complained of mild left iliac fossa pain and constipation, of which the relation to the underlying malignancy is uncertain, but she remained systemically well throughout.

The patient underwent diagnostic laparoscopy in July 2022, where a mass was seen apparently arising from the distal pancreas and abutting onto the spleen and the greater curvature of the stomach but without obvious peritoneal disease. The procedure was subsequently converted to open in the form of distal pancreatectomy and splenectomy through a left subcostal incision. A wedge gastrectomy was also performed in this case, given the apparent involvement of the stomach. Surgery was challenging as a result of adhesions from previous cholecystectomy and appendicectomy. The specimen was removed en bloc, and a Robinson drain was placed in the left upper quadrant. Postoperatively, the patient was monitored in the intensive care unit for three days, and after an uneventful recovery, was discharged home following drain removal on day eight with pancreatic enzyme supplements together with immunisations and lifelong phenoxymethylpenicillin as post-splenectomy prophylaxis. At her latest follow-up in May 2023, she was clinically well and had returned to her usual activities, including swimming, but remained slightly anxious about the future. She is still alive at the time of writing and will have surveillance imaging in September later this year.

**Figure 2:**
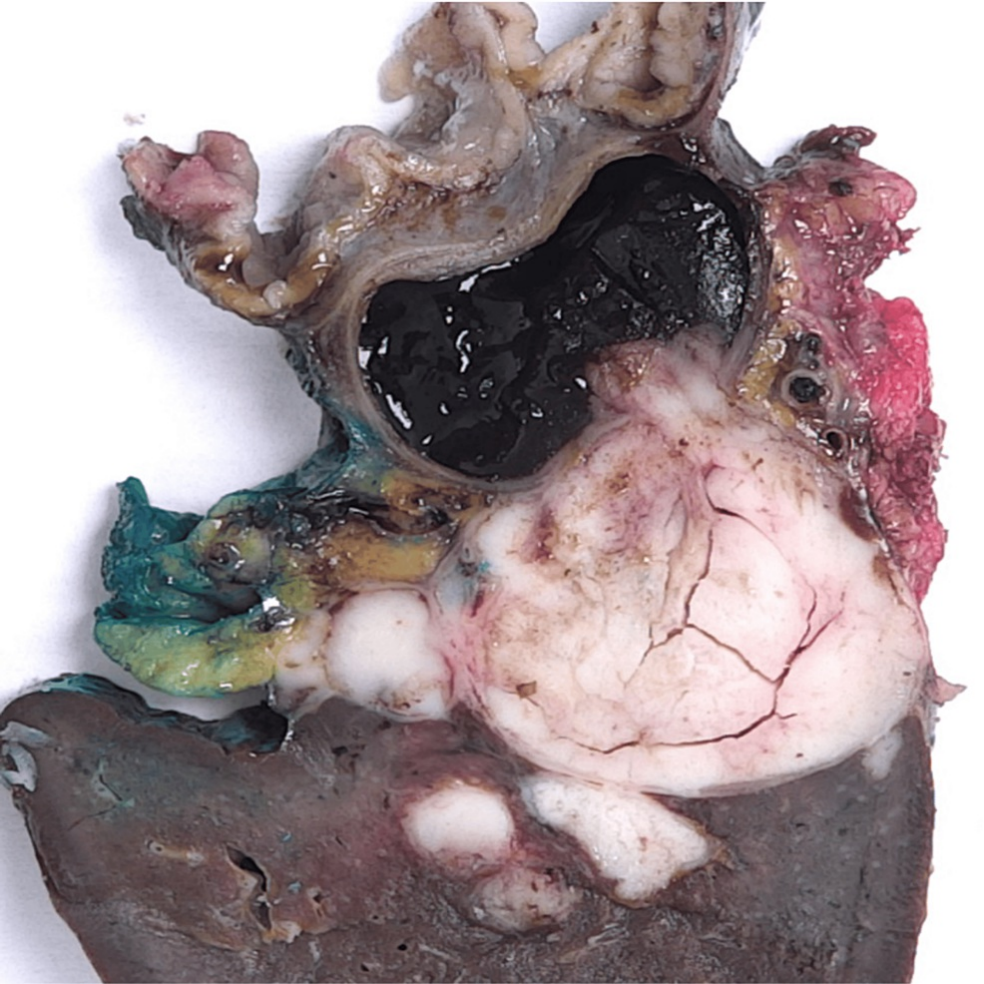
Cross-section of the resected specimen. The tumour appears confined to the spleen with both solid and cystic components and a relatively smooth interface with the splenic parenchyma.

The dimensions of the resected spleen were 13 cm × 7 cm × 4 cm. The splenic hilum contained a large tumour conglomerate measuring 5.8 cm in maximum dimension, which was partly cystic and contained a thick, violaceous, gelatinous material. The cystic component measured up to 3.5 cm × 2.5 cm × 2.5 cm and was multilocular, with one locule extending into the hilar region. It was well-circumscribed and well-demarcated from the rest of the splenic parenchyma (Figure 2). 

**Figure 3:**
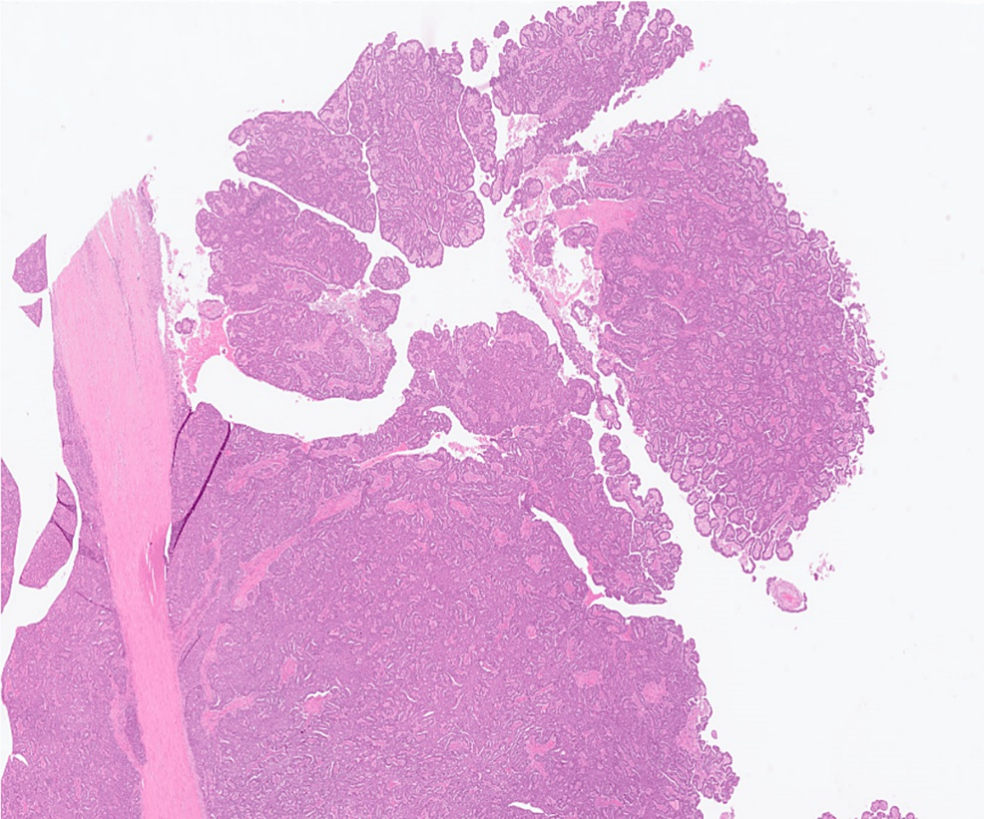
Solid-cystic tumour with papillary architecture and focal hierarchical branching. Hematoxylin and eosin (H&E) staining, original magnification ×12.

Histology demonstrated a predominantly circumscribed but focally infiltrative solid-cystic tumour localised to the splenic parenchyma, adjacent to the hilum. The tumour had a predominantly papillary and focal tubular architecture, with hierarchical branching of papillae. The neoplastic cells were low columnar to cuboidal with moderate to marked patchy nuclear pleomorphism. No cytoplasmic mucin was identified (Figure 3). These cytoarchitectural features were reminiscent of an ovarian or pancreatic serous cystadenocarcinoma. There was a tumour-free fat plane between the pancreatic tail and spleen, and the pancreatic parenchyma was free of neoplasia. The mass reached but did not infiltrate the gastric wall, and the excision was complete (R0). Venous invasion was identified.

**Figure 4:**
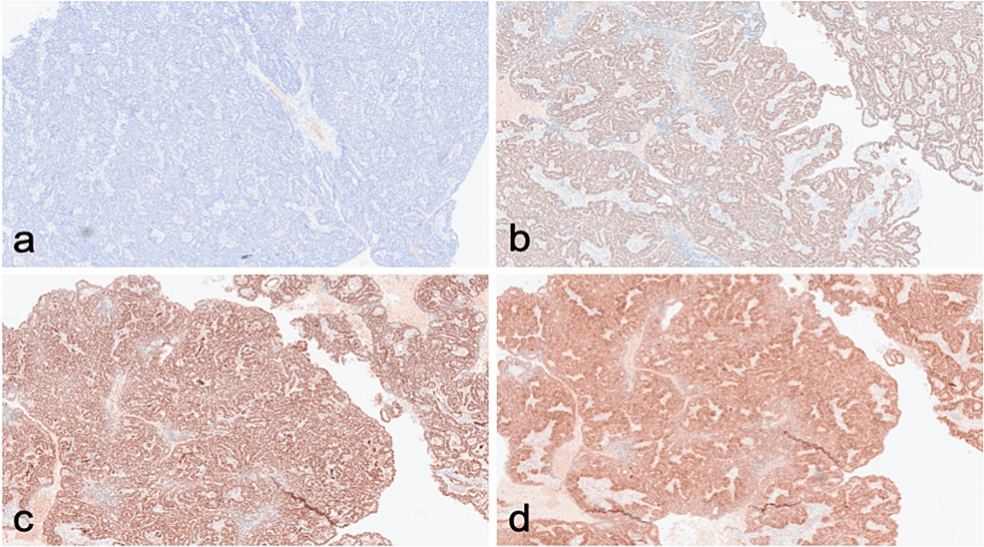
The neoplastic cells stained negative for CDX2 (a) but positive for WT-1 (b), ER (c), and PAX-8 (d). Immunohistochemistry, original magnification ×50.

The neoplastic cells were diffusely positive for pancytokeratin, CK7, CK19, CA 125, PAX8, WT1, and ER; and negative for CDX2, CEA, chromogranin, synaptophysin, CD34, CK5/6, CK20, desmin, inhibin, and p53 (Figure 4). This immunoprofile was in keeping with the morphological suggestion of serous differentiation of the carcinoma rather than mucinous. Serum CEA, CA 19-9, and CA 125 were measured in October 2022, and the levels were within normal limits.

## Discussion

Histologically, the splenic parenchyma is composed of lymphoid and vascular tissues, neither of which has a serous-secreting function [5]. Since normal splenic tissue lacks proper epithelial components, five hypotheses have been suggested to explain the histogenesis of primary cystadenocarcinomas of the spleen: heterotopic pancreatic tissue; heterotopic intestinal tissue; invaginated mesothelium of the splenic capsule; local invasion from pancreatic malignancy; and metastasis [6].

The incidence of pancreatic heterotopy, or the presence of pancreatic tissue outside its normal location without anatomic or vascular continuity with the pancreas, ranges from 0.55% to 13.7% in postmortem studies [7]. It usually occurs along the upper gastrointestinal tract, and only 1% of cases have been detected in the spleen [4]. Although predominantly asymptomatic, pancreatic rests rarely undergo the same pathological changes as normal pancreas, including malignant transformation [8]. To conclude that a tumour developed from a pancreatic rest, the following conditions must be fulfilled: first, normal pancreatic tissue should be present in the organ together with a neoplastic component, and, if possible, a transition should be histologically proven between them; and second, a neoplastic lesion should not be found in the pancreas [4]. Most recorded cases of mucinous cystadenocarcinoma of the spleen were attributed to this phenomenon (Table 1). Histological and immunohistochemical examination of our specimen did not reveal any evidence of pancreatic exocrine or endocrine tissue. 

**Table 1:** Cases of primary splenic cystadenocarcinoma recorded in the literature.

Citation	Demographics	Presentation	Management	Proposed origin
Sciberras et al. (this case)	83 ♀, United Kingdom (UK)	Incidental finding	Distal pancreatectomy, partial gastrectomy and splenectomy	Malignant transformation of an epidermoid cyst
Wlaźlak et al. [1]	45 ♀, Poland	Incidental finding	Splenectomy	Unknown
Raul et al. [9]	61 ♂, India	Abdominal pain and distension	Splenectomy	Malignant transformation of an epidermoid cyst
Fujino et al. [10]	63 ♂, Japan	Abdominal distension	Splenectomy and distal gastrectomy	Heterotopic pancreatic tissue
Ohe et al. [11]	66 ♀, Japan	Abdominal pain	Splenectomy and adjuvant chemotherapy	Invaginated mesothelium of the splenic capsule
Shiono et al. [2]	74 ♀, Japan	Unknown	Splenectomy	Unknown
Nisar et al. [8]	69 ♀, UK	Abdominal pain and breathlessness	Splenectomy and adjuvant chemotherapy	Heterotopic pancreatic tissue
Hirota et al. [6]	68 ♀, Japan	Incidental finding	Splenectomy and left nephrectomy, followed by delayed distal pancreatectomy	Heterotopic pancreatic tissue
Morinaga et al. [12]	69 ♂, Japan	Abdominal mass	Splenectomy	Invaginated mesothelium of the splenic capsule
Zanetti et al. [13]	21 ♀, Italy	Abdominal pain	Splenectomy and adjuvant chemotherapy	Heterotopic pancreatic tissue
Matsumoto et al. [14]	69 ♂, Japan	Abdominal pain, vomiting and constipation	Splenectomy, distal pancreatectomy and left hemicolectomy	Invasion from malignancy of the tail of pancreas
Shuman and Bouterie [15]	42 ♀, United States of America (USA)	Abdominal mass	Splenectomy and distal pancreatectomy	Heterotopic pancreatic tissue

Miracco and colleagues reported a case of a splenic cyst lined with mucus-secreting epithelium, which they believed to be of intestinal origin due to positive CEA staining [16]. Pancreatic heterotopy would also exhibit this immunohistochemistry finding, but the authors did not consider this possibility.

Cysts of the spleen are divided into true (primary or epithelial-lined) and false (secondary or pseudocysts) cysts. Epidermoid cysts (metaplastic mesodermal cysts), the commonest true cyst of the spleen, are lined with squamous epithelium [12]. In other instances, the epithelium is cuboidal to low columnar, resembling the 'mesothelial' lining. The exact histogenesis of primary cysts remains inconclusive, but dysembryological origins from adjacent organs or traumatic invagination of the capsular surface mesothelium with subsequent cystic expansion have been proposed [14]. Epidermoid cysts have tumorigenic potential, undergoing metaplasia and dysplasia to develop into squamous cell carcinoma and mucinous cystadenocarcinoma. Malignant transformation of cysts with a similar mesothelial or serous phenotype has been well documented in the ovary and pancreas [17,18]. 

Our tumour was positive for CK7, ER, and WT1 expression and negative for CK20 and CEA, which is consistent with serous cystadenocarcinoma [19] and virtually excludes the possibility of mucinous tumours and pancreatic or gastrointestinal malignancy [20]. Our immunohistochemistry panel would also be in keeping with endometrial or ovarian neoplasms [19]; however, after an alternative malignancy was ruled out clinically, biochemically, and radiologically, our opinion is that this is a case of serous cystadenocarcinoma of the spleen developing within a benign epidermoid cyst and the first of its kind in the literature.

One other possible case was reported in a 65-year-old Chinese female who also developed cervical lymph node metastases. She underwent resection of a smooth-walled multilocular cystic tumour that lacked mucin-secreting epithelium. The tumour cells stained positive for CK-7 and Ber-EP4 but were negative for CK-20, CDX2, and TTF-1. The authors concluded that the tumour had arisen either from early ovarian bloodborne micro-metastases or occult cancer cells, which were released from normally appearing ovaries during total abdominal hysterectomy and bilateral salpingo-oophorectomy nine years prior. The patient had elevated serum levels of CA 125 and CA 15-3 preoperatively, which normalised after surgery. This observation, together with the immunohistochemistry results and normal serum levels of CEA and CA 19-9, reinforced their claim and decreased the likelihood that the tumour had gastrointestinal or pancreatic origins [5].

## Conclusions

Cystadenocarcinomas necessitate total curative excision, and inept biopsy or rupture may cause dissemination and preclude the possibility of cure. The uniqueness of this case also created uncertainty around adjuvant treatment options, particularly in the event of local or distal recurrence, as well as the optimal method of follow-up. We offered the patient a treatment regimen similar to that used in serous cystadenocarcinoma of the pancreas, with radical surgery followed by adjuvant chemotherapy, which she declined. She has remained well without evidence of recurrence or metastasis and will be kept under regular follow-up with yearly CT scans and clinical assessment.

Primary splenic malignancy is an extremely rare diagnosis. We recommend taking a multidisciplinary approach to such cases with heavy patient involvement in the decision-making after a thorough discussion of the risks and benefits of the different management options.
